# Potent Inhibition of Hendra Virus Infection via RNA Interference and Poly I:C Immune Activation

**DOI:** 10.1371/journal.pone.0064360

**Published:** 2013-05-14

**Authors:** Jana L. McCaskill, Glenn A. Marsh, Paul Monaghan, Lin-Fa Wang, Timothy Doran, Nigel A. J. McMillan

**Affiliations:** 1 The University of Queensland Diamantina Institute, Brisbane, Queensland, Australia; 2 CSIRO Livestock Industries, Australian Animal Health Laboratory, Geelong, Victoria, Australia; 3 Griffith Health Institute and School of Medical Science, Griffith University, Southport, Queensland, Australia; University of Tennessee Health Science Center, United States of America

## Abstract

Hendra virus (HeV) is a highly pathogenic zoonotic paramyxovirus that causes fatal disease in a wide range of species, including humans. HeV was first described in Australia in 1994, and has continued to re-emerge with increasing frequency. HeV is of significant concern to human health due to its high mortality rate, increasing emergence, absence of vaccines and limited post exposure therapies. Here we investigate the use of RNA interference (RNAi) based therapeutics targeting HeV in conjunction with the TLR3 agonist Poly I:C and show that they are potent inhibitors of HeV infection *in vitro*. We found that short interfering RNAs (siRNAs) targeting the abundantly expressed N, P and M genes of HeV caused over 95% reduction of HeV virus titre, protein and mRNA. Furthermore, we found that the combination of HeV targeting siRNA and Poly I:C had an additive effect in suppressing HeV infection. Our results demonstrate for the first time that RNAi and type I interferon stimulation are effective inhibitors of HeV replication *in vitro* and may provide an effective therapy for this highly lethal, zoonotic pathogen.

## Introduction

Hendra virus (HeV) is an emerging zoonotic virus of the family *Paramyxoviridae* that is distinguished by its ability to cause fatal disease in both human and animal hosts. HeV was first identified in the suburb of Hendra in 1994 following an outbreak of severe respiratory disease occurring in horses. Thirteen horses died during the outbreak and two humans became infected, with one fatality from an encephalic illness [1]. There have been 42 total outbreaks of HeV in Australia, with 18 events in the year 2011 alone, resulting in the death of 21 horses [2]. Seven human cases of HeV have been confirmed, with only 3 patients recovering from infection. The closely related Nipah virus (NiV) has caused many outbreaks since 1999 in South East Asia, resulting in 570 reported cases of NiV infection in humans, of which 305 where fatal (reviewed in [3]). Infection is characterised by systemic spread, with evidence of infection occurring in multiple organ systems. Widespread endothelial cell infection, vasculitis and CNS infection are key indicators of fatal disease progression and appear to be central to the pathogenesis of the disease. Together, henipaviruses (HeV and NiV) have a combined fatality rate of over 53%. These viruses are a significant concern to human health due to their high mortality rate, increasing emergence, and absence of vaccines or effective therapeutics. Recently, an antibody therapy has been reported to protect monkeys from fatal infection if given within 3 days. However, later treatment failed to prevent neurological symptoms suggesting early intervention is critical [4].

The henipaviruses are enveloped single-stranded RNA viruses of negative polarity with a genome of approximately 18.2 kb. The genome encodes six major structural proteins; the nucleocapsid (N), phosphoprotein (P), matrix (M), fusion (F), glycoprotein (G), and large (L) protein or RNA polymerase. The P gene of henipavirus additionally encodes for C, V and W proteins which have been implicated in interfering with the host innate immune system, through inhibition of the interferon responses [5], [6]. Both henipaviruses have emerged from their wildlife reservoir, *Pteropus* spp. fruit bats, in the past 15 years. Infection of humans occurs via intermediate amplifying hosts. For HeV, horses have served as the intermediate [7], [8] while NiV has been shown to infect via pigs [9]. However, human NiV outbreaks in India and Bangladesh have resulted from direct bat to human transmission, with contaminated raw date palm sap a likely source for several outbreaks [10], [11].

A potential therapeutic for HeV infection is RNA interference (RNAi). RNAi is a natural cellular viral defence pathway in which dsRNA sequences are used to degrade homologous target mRNA and has shown promise in the clinical setting, with multiple therapeutics targeting human diseases currently in development [12]. The major advantages of RNAi are high target specificity, the ability to silence virtually any gene (or multiple genes), protection of uninfected cells, and inhibition of viral replication in infected cells. This compares to antibody therapies that can only target free virus. Current clinical trials of RNAi-based therapeutics include treatments against several viral diseases, such as Hepatitis B [13] and Respiratory syncytial virus [14]. We have previously shown that henipaviruses are susceptible to inhibition via RNAi [15]. This study investigated the efficiency of RNAi against NiV and HeV *in vitro*, and showed that targeting the abundantly expressed, early transcript N, was superior compared to targeting the later, less abundant L transcript.

Given the potential of RNAi and the unmet clinical need we investigated the use of siRNAs to treat HeV infection as a forerunner to *in vivo* studies. Here we present data on the use of RNAi to target a range of the early transcribed, abundantly-expressed HeV genes (N, P and M) and identify several highly potent siRNAs that reduce gene expression, protein levels and viral replication. Additionally, we examined the potential of targeting multiple viral genes as well as the use of multiple siRNAs targeting the same viral gene. Finally, we tested pre-treatment’s of siRNAs combined with the potent toll-like receptor 3 (TLR3) and RIG-like receptor (RLR) agonist, Polyinosinic:polycytidylic acid (Poly I:C) as a potential bi-functional therapy.

## Results

### siRNA Selection and Performance

HeV transcription is initiated from the 3′ terminus of the genomic RNA and the relative abundance of each viral transcript relates to its distance from the 3′ terminus of the genome [16]. For example, mRNA encoded by genes such as the nucleoprotein (N) and phosphoprotein (P) accumulate at much higher levels than those transcribed from the large polymerase (L) gene. These highly abundant genes are also critical for infection and thus targeting them via RNAi may be more effective. Previous work supports this hypothesis with an N targeting siRNA showing more potency at silencing HeV than siRNAs targeting L [15]. Therefore we designed a range of potential siRNAs targeting the N, P and M genes. In total 16 HeV specific siRNAs were designed; eight sequence-specific siRNAs targeting the N gene, four targeting the P gene, and four targeting the M gene. We used the previously published sequences of HeV N specific siRNAs in addition to newly designed siRNAs against N, P and M (Table 1).

**Table 1:** Sequence characterisation of HeV siRNAs and D-siRNAs targeting N, P and M genes.

Identifier	Sense 5′–3′	Antisense 5′–3′
siN4	CUGAAGACUGCACGUGAAAUU	UUUCACGUGCAGUCUUCAGUU
siN5	GGACUGAGGAUCACCGAUAUU	UAUCGGUGAUCCUCAGUCCUU
siN6	AGAGGGUCAAUCCAUUCUUUU	AAGAAUGGAUUGACCCUCUUU
siN7	GGTAAAGAAAGGCGGAUCAUU	UGAUCCGCCUUUCUUUACCUU
siN8	AGGAAAUUAUGUCGAGGAAUU	UUCCUCGACAUAAUUUCCUUU
siN11	ACAUCAUGCUGGCGGGAUUUU	AAUCCCGCCAGCAUGAUGUUU
siN12	CAUGCAGGCAAGAGAAGCCUU	GGCUUCUCUUGCCUGCAUGUU
siN15	GCACAGAGCUCAUCGGAAAUU	UUUCCGAUGAGCUCUGUGCUU
siP2	CAACCAAGUACCAAAGACAGGACaa	UUGUCCUGUCUUUGGUACUUGGUUGUU
siP8	GGAGUAUGAGGAUGAGUUUGCCAaa	UUUGGCAAACUCAUCCUCAUACUCCAG
siP10	AGAAGAAACUCCUGAUGUACGCAga	UCUGCGUACAUCAGGAGUUUCUUCUUU
siP18	AGGCAAGGGUGAAAGGAAAGGGAaa	UUUCCCUUUCCUUUCACCCUUGCCUUU
siM2	GCCAGAAAUUGAUAAGCAUGGCAgt	ACUGCCAUGCUUAUCAAUUUCUGGCUC
siM5	CUCUUACCAUGGAAGAAGAUUCUga	UCAGAAUCUUCUUCCAUGGUAAGAGAU
siM7	CGACAAAGACGGAACCAAAGUGGca	UGCCACUUUGGUUCCGUCUUUGUCGAA
siM8	AGAGAAAGAUUGACAGAAUGAAGct	AGCUUCAUUCUGUCAAUCUUUCUCUUG
ScrM7	CGUUAAUCGCGUAUAAUACGCGUat	AUACGCGUAUUAUACGCGAUUAACGAC

Nucleocapsid siRNAs are conventional 21 bp siRNAs. All M, P and ScrM7 are D-siRNAs that are 25+27 bp long, with a two RNA nucleotide overhang only on the antisense strand and two DNA nucleotides on the sense strand depicted in lowercase letters.

**Figure 1:**
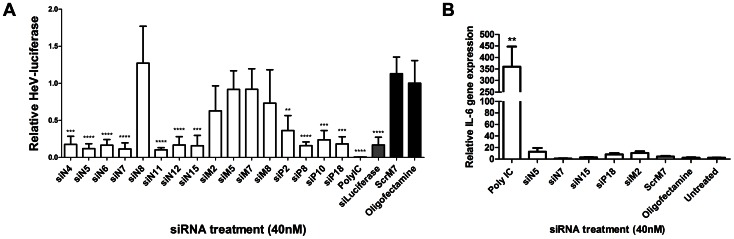
Ability of siRNAs to knockdown recombinant HeV-luciferase and cause immune stimulation. HeLa cells were transfected with 40 µg/ml Poly IC, 40 nM control or HeV targeting siRNAs. siLuciferase was a positive control (grey bar), while scrambled (ScrM7) siRNA and Oligofectamine were negative controls (black bars). **A)** Transfection media was replaced after 4 hours and cells were infected with recombinant HeV-luciferase at an MOI of 0.5. Infected cells were incubated for 24 hours before luciferase activity was measured. Levels of luciferase luminescence were normalized to Oligofectamine control levels and are the mean ± S.D. of six biological replicates from two independent experiments. **B)** Cells were incubated for 12 hours before RNA extraction. Gene expression levels of IL-6 were quantified by qRT-PCR as relative expression to B-actin housekeeping gene. Results are the mean ± S.D. of six biological replicates from two independent experiments. Significant differences between Oligofectamine control and siRNAs are indicated (**p = <0.01, ***p = <0.001, ****p = <0.0001; two-sided t-test).

Initial experiments evaluating the siRNA efficacy were performed using the human cervix carcinoma cell line, HeLa-CCL2, which supports both HeV replication and RNAi mediated gene silencing, thus providing a model cell to assess RNAi mediated HeV reductions in human cells. HeLa cells were infected with a recently developed recombinant HeV encoding a firefly luciferase (LUC) reporter inserted between the P and M genes [17]. Here luciferase is expressed as an additional gene insert in the virus genome and luciferase levels are proportional to virus replication. Luciferase expression therefore acts as a surrogate marker of HeV replication and thus enabled the preliminary screening of the HeV-specific siRNAs in a rapid manner using minimal laboratory labour. These are important factors to consider when conducting experiments with a BSL4 pathogen. The recombinant HeV-luciferase system was used to screen all sixteen siRNAs targeting the N, P or M genes of HeV by transfecting cells with siRNA 4 hours prior to infection with recombinant HeV (MOI 0.5). An siRNA concentration of 40 nM was utilized as this was comparable to the 2 mg/kg treatment employed for *in vivo* RNAi experiments [18], [19]. Infected cells were incubated for 24 hours before luciferase activity was measured. The majority of siRNAs targeting the N and P genes caused highly significant reductions in LUC expression with the exception of siN8 and to a lesser extent siP2 (Figure 1a). This level of reduction (75–90%) was similar to that caused by the positive control siRNA, siLuciferase (Dharmacon), which directly targeted the LUC mRNA. Poly I:C treatment also resulted in potent (99%) reductions in LUC expression. However the four siRNAs targeting the M gene did not cause any significant knockdown of luciferase expression. The lack of luciferase silencing exhibited by M targeting siRNAs was an expected result. HeV can spread either via budding virus or fusion of neighbouring cells. Knock-down of M gene will prevent budding virus but not cell-to-cell spread via fusion. At an MOI of 0.5, virus will spread efficiently to all cells within 24 hours via cell fusion, accounting for the lack of an effect of LUC levels.

This screen allowed us to identify lead candidate siRNAs for further investigation. Three siRNAs targeting N were selected; siN5 (∼90% inhibition), siN7 (∼90% inhibition) and siN15 (∼85% inhibition). Additionally, the best P and M targeting siRNAs were selected, siP18 (∼80% inhibition) and siM2 (∼35% inhibition).

Considering the potent reduction of recombinant HeV seen from treatment with Poly I:C in our initial screen one may argue that the reductions from siRNA treatment were caused by non-specific immune effects. Therefore, our lead candidate siRNAs were analysed for the capability to cause immune activation as measured by increased expression of the pro-inflammatory cytokine IL-6 (Figure 1b). While Poly I:C treatment caused significant upregulation of IL-6, none of the lead candidate siRNAs stimulated significant IL-6 expression when compared to controls. As none of the lead candidate siRNAs were inherently immunostimulatory they were analysed further for suppression of HeV replication *in vitro*.

### Reduction in Viral Titres

**Figure 2:**
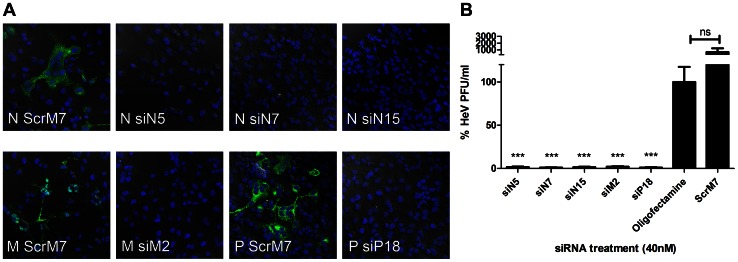
Reductions in HeV via pre-treatment with RNAi. HeLa cells were transfected with 40 µg/ml Poly IC, 40 nM control or HeV targeting siRNAs. Transfection media was replaced after 4 hours and cells were infected with HeV clinical isolate at an MOI of 0.1. Infected cells were incubated for 24 hours before **A)** Infected cells were fixed and labelled for HeV N, P or M protein and DAPI stain. Cells were imaged with a Leica confocal microscope. Indicative images are shown. Scale bar = 50 µM. **B)** Supernatant was removed and titrated for TCID_50_ assay. Cells infected with supernatant were incubated for 3 days before TCID_50_ was calculated and shown as the mean ± S.E.M. of six biological replicates from two independent experiments. Significant differences between Oligofectamine control and siRNAs are indicated (***p = <0.001, two-sided t-test).

The initial screen above used a surrogate marker of HeV gene expression. The next step was to elucidate the efficiency of our selected HeV-specific siRNAs targeting N, P and M using live viral infections assays. HeV protein expression of siRNA-treated, virally infected cells was analysed via confocal microscopy using target-specific antibodies. We observed potent reductions in N, P and M protein expression following a single siRNA pre-treatment, when compared to the negative control scrambled siRNA (Figure 2a).

To further confirm that the siRNA reductions in protein expression resulted in the suppression of HeV replication, we measured total virus output. HeLa cells were transfected for 4 hours with 40 nM siRNAs prior to infection with a clinical isolate of HeV before virus replication was determined at 24 hours by TCID_50_. In agreement with the luciferase assays above, we observed a >98% (1.5 log) reduction in HeV replication when cells were pre-treated with the N and P siRNAs as indicated by a reduction in virus titre (Figure 2b). As expected, the siRNA targeting M, siM2, which had shown minimal silencing of the recombinant HeV, caused significant reductions in release of infectious HeV (1.4 log; 97% inhibition). Pre-treatment with scrambled siRNA (ScrM7) resulted in no significant loss of viral replication.

### Combined Pre-treatments

**Figure 3:**
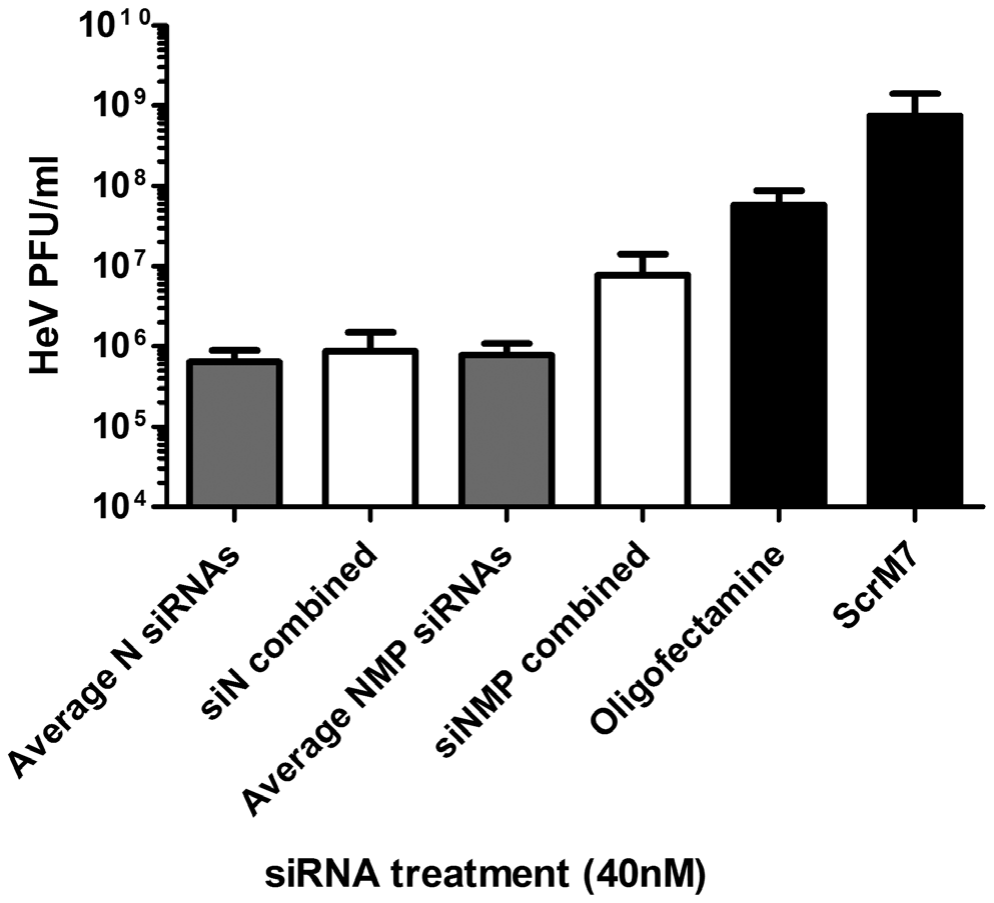
Reductions in HeV titre via pre-treatment with combined RNAi. HeLa cells were transfected with 40 nM combined HeV targeting (white bars) or control (black bars) siRNAs. Transfection media was replaced after 4 hours and cells were infected with HeV clinical isolate at an MOI of 0.1. Infected cells were incubated for 24 hours before supernatant removed and processed for TCID_50_ assay. Cells infected with supernatant were incubated for 3 days before TCID_50_ calculation and are shown as the mean ± S.E.M. of six biological replicates from two independent experiments. Differences between the combination treatments and expected values based on the average of singular siRNA treatments (grey bars) are shown.

As the use of siRNAs targeting only a single HeV gene appeared to be highly effective in reducing HeV replication we hypothesized that combining these potent single siRNAs could have additional benefits in silencing HeV. To test this hypothesis we investigated two approaches. The first was a combination of the three siRNAs targeting the N gene (siN5, siN7 and siN15 at a 1∶1∶1 ratio) at a final concentration of 40 nM. The second approach was a combination of the three most potent siRNAs targeting each gene; siN15, siM2 and siP18, once again at 40 nM final concentration with equal ratios of each siRNA. As before, HeLa cells were transfected with siRNAs and at 4 hours HeV infection undertaken and reductions in HeV replication measured after 24 hours via 50% Tissue Culture Infective Dose (TCID_50_) endpoint dilution assay. The reductions in virus titre were compared to the average silencing achieved by the individual siRNA pre-treatments (Figure 3). Surprisingly, the results showed that neither of the combination pre-treatments provided any additional suppression of HeV replication. The combined N siRNA pre-treatment (siN combined) had similar levels of HeV silencing when compared to the single average levels. However, the N, P and M combined siRNA pre-treatment (siNMP combined) had reduced silencing potency when compared to the expected level of knockdown.

**Figure 4:**
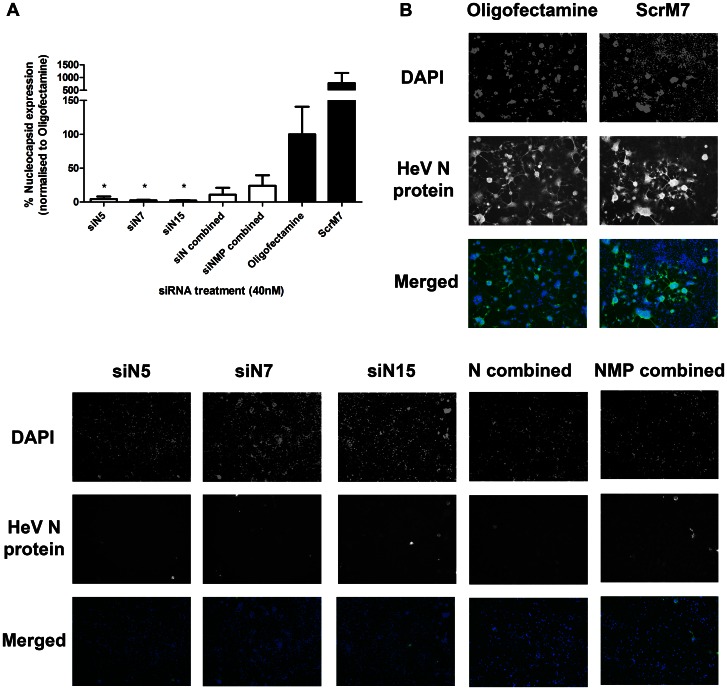
Reductions in HeV gene expression and protein caused by RNAi targeting HeV. HeLa cells were transfected with 40 µg/ml Poly IC, 40 nM control or HeV targeting siRNAs. Transfection media was replaced after 4 hours and cells were infected with HeV clinical isolate at an MOI of 0.1. **A)** Infected cells were incubated for 24 hours before RNA extraction. Gene expression levels of N were quantified by qRT-PCR as relative expression to B-actin housekeeping gene. Results are the mean ± SEM of six biological replicates from two independent experiments. Significant differences between Oligofectamine control and siRNAs are indicated (*p = <0.05; two-sided t-test). **B)** Infected cells were incubated for 24 hours and were subsequently fixed and labelled for HeV N protein and DAPI nuclear stain. Cells were imaged with an EVOS Microscope at 10x objective and analysed for the presence or absence of GFP indicating N protein. Indicative images are shown.

One explanation for this outcome is that in combination pre-treatments each siRNA was present at one third the original concentration and thus was not as potent at silencing. In order to understand why the combined pre-treatments were not as effective as single treatment we examined the levels of gene expression, rather than viral output. Therefore, reductions in HeV gene expression caused by singular and combination siRNA pre-treatments were measured via qRT-PCR (Figure 4a). Significant reductions in N gene levels were seen upon pre-treatment with N-specific siRNAs but not controls. While the singular siRNAs caused over 95% inhibition of N mRNA, the N and NMP combination pre-treatments did not result in significant N silencing. This confirmed that the combinations were not as effective in reducing gene expression, which is presumably due to the fact that each individual siRNA is present at a third the original level in order to keep the overall siRNA dose constant.

We further examined the levels of protein expression caused by pre-treatment with singular and combination siRNAs via immunofluorescence microscopy. RNAi-treated cells were infected with HeV and the level of N protein expression was analysed after 24 hours (Figure 4b). Immunofluorescence microscopy analysis indicated potent knockdown of N protein by pre-treatment with single siRNAs. While significant reductions in N protein expression were also seen with combination siRNA pre-treatments, this was not to the level of singular siRNA pre-treatment.

### Poly I:C TLR3 and RLR Activation Enhances siRNA Suppression

**Figure 5:**
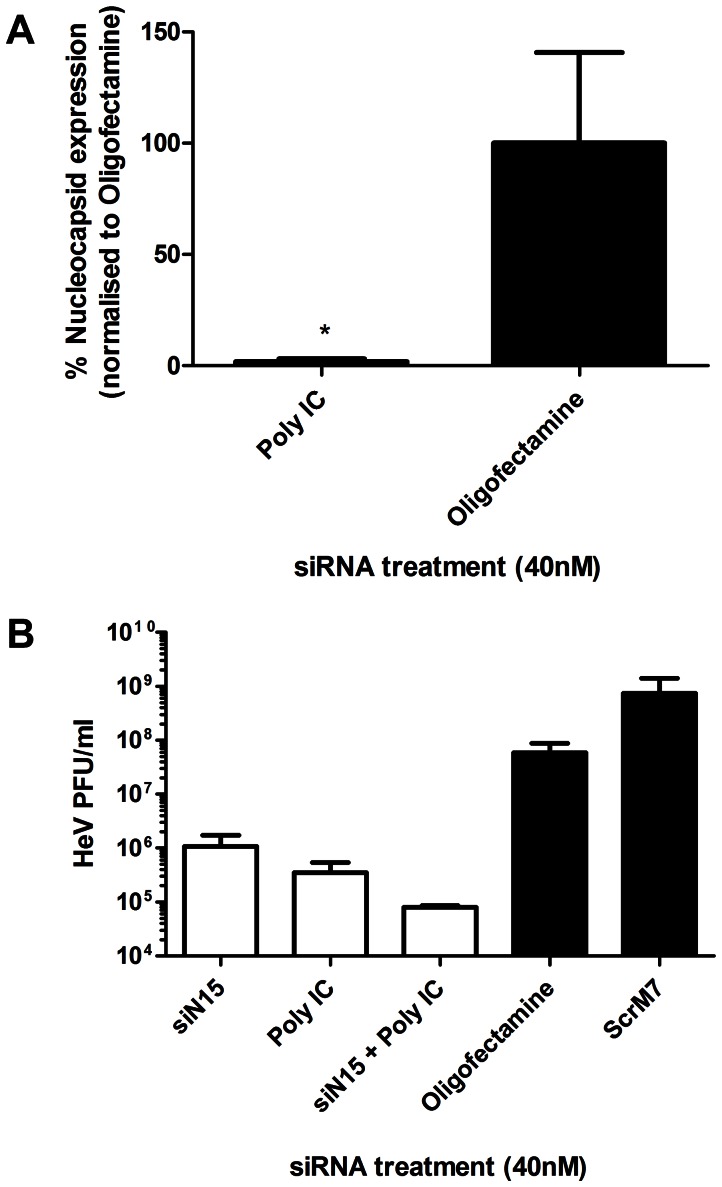
Reductions in HeV titre via pre-treatment with RNAi and Poly IC. HeLa cells were transfected with 40 µg/ml Poly IC, 40 nM control (black bars), HeV targeting siRNA, or combined Poly IC/siN15. Transfection media was replaced after 4 hours and cells were infected with HeV clinical isolate at an MOI of 0.1. **A)** Infected cells were incubated for 24 hours before RNA extraction. Gene expression levels of N were quantified by qRT-PCR as relative expression to B-actin housekeeping gene. Results are the mean ± SEM of six biological replicates from two independent experiments. Significant differences between Oligofectamine control and siRNAs are indicated (*p = <0.05; two-sided t-test). **B)** Infected cells were incubated for 24 hours before supernatant removed and processed for TCID_50_ assay. Cells infected with supernatant were incubated for 3 days before TCID_50_ calculation and are shown as the mean ± S.E.M. of six biological replicates from two independent experiments.

TLRs and RLRs have been shown to induce the expression of cytokines known to have potent antiviral activity such as the type I interferon’s (IFN). As IFNs have previously been shown to be antagonised in HeV infections [20] we wondered if IFN agonists would be useful in a combined therapy setting, particularly if they are delivered in the same manner as siRNAs via liposomes. Poly I:C is a synthetic mimetic of dsRNA that is known to interact with cell surface and endosomal TLR3 or with cytoplasmic RNA helicases such as melanoma differentiation-associated gene 5 (MDA5) and retinoic acid-inducible gene I (RIG-I) upon transfection into cells [21], [22]. Preliminary testing of Poly I:C in the recombinant HeV luciferase assay had indicated that it was a potent suppressor of HeV replication (Figure 1a). Therefore Poly IC was further investigated for the ability to inhibit N mRNA (Figure 5a). Poly IC was seen to significantly inhibit HeV N mRNA when compared to Oligofectamine control.

Poly I:C and the singular HeV specific siRNAs pre-treatments consistently resulted in high levels of mRNA, protein and virus titre knockdown. We therefore performed further analyses of these HeV pre-treatments by combining Poly I:C and siN15, a potent silencing siRNA targeting HeV N gene. Cells were treated with single and combined siN15 and Poly I:C and were subsequently infected with HeV. After 24 hours, suppression of virus replication was measured via virus titre reductions (Figure 5b). Poly I:C and siN15 individual pre-treatments resulted in a 1.5 log (<95% reduction) of HeV. The combination siN15-Poly I:C pre-treatment resulted in an over 2.5 log (<99.9% reduction) in virus titre, indicating that there is a trend for the two pre-treatments to act simultaneously to cause additional suppression of HeV.

**Figure 6:**
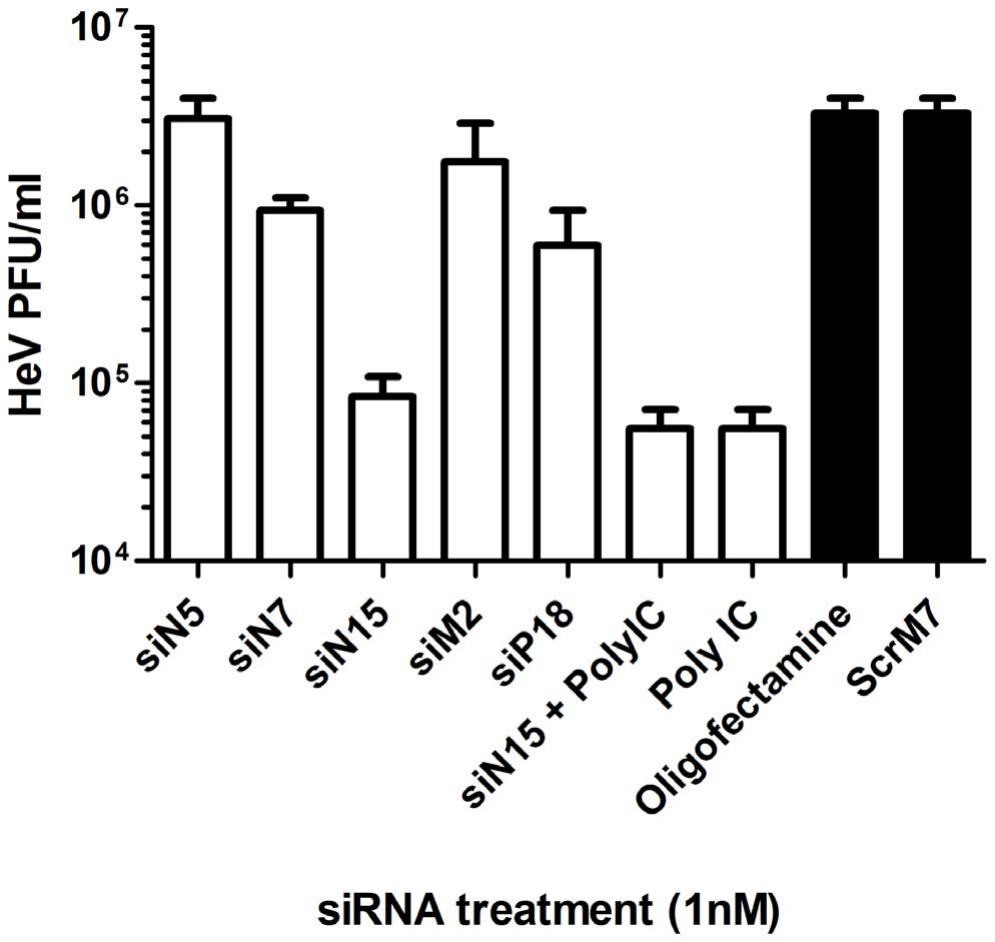
Reductions in HeV titre via treatment with lower RNAi concentrations. HeLa cells were transfected with 1 µg/ml Poly IC, 1 nM of control or HeV targeting siRNAs. Transfection media was replaced after 4 hours and cells were infected with HeV clinical isolate at an MOI of 0.1. Infected cells were incubated for 24 hours before supernatant removed and processed for TCID_50_ assay. Cells infected with supernatant were incubated for 3 days before TCID_50_ calculation and are shown as the mean ± S.E.M. of three biological replicates.

Due to the high silencing potency of the majority of siRNAs targeting HeV, an additional experiment was undertaken to determine the effectiveness of these siRNAs to suppress HeV when transfected into cells at lower concentrations. Cells were treated with a 40× lower dose (i.e. 1 nM) of specific siRNAs or Poly I:C and subsequently infected with HeV. Suppression of HeV replication was measured via virus titre 24 hours post infection (Figure 6). While the majority of the single siRNA pre-treatments had reduced silencing potency at 1 nM concentration, siN15, Poly I:C, and the combination of siN15-Poly I:C still resulted in 1.5 log (<95% reduction) in virus titre even at this low dose.

## Discussion

Henipaviruses remain a significant global threat due to the high number of susceptible species, the wide *Pteropus* spp. reservoir host range and high mortality. The development of RNAi as a novel antiviral to assist in protection against HeV presents an innovative approach in managing a zoonotic virus outbreak. Given the fact that recent Nipah virus outbreaks in Bangladesh exhibit human-to-human transmission with a mortality rate of 75% [23] it is clear that the Henipavirus genus represents a significant health threat [2]. Results presented here clearly showed that cells treated with siRNAs targeting the 3′ abundantly expressed genes of HeV had a highly potent sequence-specific anti-viral response with reductions in virus titre in the order of 3.5 logs (99.95%). Additionally, we show that testing of combination RNAi targeting HeV provided no additional benefits to efficient, single, siRNA pre-treatments. Furthermore, the use of TLR3 and RLR agonist, Poly IC, in combination with single siRNA pre-treatment showed additional advantages when compared to either treatment alone. To our knowledge, this is the first paper that describes the use combinational RNAi and Poly I:C for treatment of HeV.

We first examined the effect of a range of HeV specific siRNAs targeting the abundantly expressed, early transcribed N, P, and M genes located at the 3′ end of the HeV genome. Initial results obtained from the recombinant HeV-luciferase assay showed that while siRNAs targeting N and P genes caused over 50% suppression of HeV, those specific for M caused minimal reductions in HeV. However, further analysis of virus titre and protein expression showed that M sequence specific siRNAs resulted in potent HeV silencing. This discrepancy may have been caused by the nature of the spread of HeV in infected cells which is not dependant on budding. Thus any changes or reductions in the other genes of the HeV genome may not result in significant reductions in luciferase expression. While the recombinant HeV luciferase assay provides a superior screening assay for a BSL4 environment, these preliminary observations should be followed up with detailed virus analysis. Our subsequent examination of sequence specific siRNAs targeting N, P and M showed that each single siRNA pre-treatment caused over 1.5 log (95%) reduction of virus titre, and a reduction of protein expression to undetectable levels. Furthermore, the 1.5 log (95%) reduction of virus titre was still evident with siN15 pre-treatment at 40X less concentration (1 nM). These results provided further evidence that the use of RNAi to target HeV is a powerful treatment option.

The use of combinational siRNA has been addressed in the literature due to the concern that RNAi mono-therapy may be susceptible to virus escape, especially from viruses such as HIV that have been shown to only require 25 days before mutational escape from RNAi [24]. However, the HeV genome is highly conserved with less than 1% sequences variation across the entire genome [25] and is thus unlikely to execute rapid mutagenesis upon RNAi mono-therapy. Therefore our study investigated the potential silencing benefits of combinational siRNA therapy targeting HeV. Interestingly, our findings indicate that combinational siRNA therapy does not hold any advantage over single siRNA pre-treatment as there was reduced knockdown of virus titre and mRNA levels caused by the siRNA cocktails. The single siRNAs used in this study had the ability to effectively suppress virus mRNA and protein levels. This would completely halt virus replication and assembly, as shown by the significantly decreased virus titres in our study. However, in the combinational pre-treatments these efficient silencing siRNAs were only a third of the single treatment concentration. This reduced siRNA concentration may diminish each silencing potential to only 30–50%. Hence, virus replication, growth and spread would still continue, though at a significantly decreased rate due to the reduction of each protein available. This may explain the decrease in silencing activity seen by the combination of siN15, siM2 and siP18 when compared to the silencing ability of each single siRNA.

A major finding from this study is the ability of Poly I:C to potently suppress HeV infection. The dsRNA structure of Poly I:C is recognised by TLR3 which activates type I IFN, inflammatory cytokine production and dendritic cell maturation [26]. The antiviral properties of type I IFNs have been well characterised with PEGinterferon used extensively for the treatment of hepatitis C virus [27]. The use of Poly I:C has previously been shown to be protective in a hamster model of NiV infection [28], but this is the first study to investigate Poly IC for the treatment of HeV. Our results showed that Poly I:C was able to cause over 2 log (99%) suppression of HeV. The susceptibility of HeV to Poly I:C treatment may be linked to the mechanisms that HeV has in place to inhibit interferon responses. The four proteins encoded by the P gene of Henipaviruses have been shown to have roles in modifying host cell immune response, by inhibiting interferon activity [5], [6], [29]–[31]. Due to the measures that Henipaviruses have taken to protect themselves from the activity of type I IFNs, it is not surprising that HeV is highly susceptible to Poly I:C treatment. These results show that pre-treatment of uninfected cells with Poly I:C can protect them from infection, suggesting that HeV infected patients may benefit from early treatment with IFN. Further studies with immune knockout cells should be undertaken to determine which pathway in the type I IFN response is responsible for HeV suppression.

We have previously shown that HeV is susceptible to silencing by RNAi through targeting of the N gene with two siRNAs that were specific for HeV N resulting in reductions of both syncytial count and viral genome [15]. In this study we have found that HeV replication is not only suppressed by targeting alternative regions of the N gene, but also through targeting the P and M genes of the virus.

In conclusion, this is the first paper describing the use of virus specific siRNA in combination with immune antagonist Poly I:C for the treatment of HeV. We showed the silencing efficiency of a range of HeV siRNAs, which result in the potent knockdown of N, P and M target gene expression. Using these siRNAs, we demonstrated that there were no benefits of combined siRNA treatment. Although further studies are required to investigate the effects of our siRNAs in an animal model of HeV infection, the potency shown by our siRNAs in suppressing HeV infection *in vitro* provides a significant advancement in the development of a HeV therapeutic. Due to the high sequence conservation shown between the two members of the Henipavirus genus, future investigations will examine the silencing potential of our siRNAs in the suppression of NiV. These HeV targeting siRNAs may also be used to investigate and further extrapolate N, P and M gene function during active replication. In addition, the approach of using RNAi to treat zoonotic viruses has many benefits as a therapeutic for new and emerging viruses. Virus specific siRNAs can be designed and tested when only the virus sequence has been confirmed, thus providing a rapid therapeutic option for the next generation of emerging zoonotic viruses.

## Materials and Methods

### siRNAs

Chemically-synthesized siRNAs were purchased from Sigma-Aldrich (Victoria, Australia). Dicer substrate siRNAs were obtained from Integrated DNA technologies (IDT; Iowa, USA). Scrambled control, ScrM7, was designed by IDT and previously published [18]. Polyinosinic-polycytidylic acid was obtained from Sigma-Aldrich.

### Cell Culture

The continuous human epithelial cell line HeLa (ATCC CCL-2) and african green monkey kidney epithelial Vero cells (ATCC CRL-81) were maintained in EMEM supplemented with 10% (v/v) heat inactivated foetal calf serum (FCS), 100 units/ml Penicillin G, 100 µg/ml Streptomycin sulphate and 2 mM L-glutamine. All cells were incubated at 37°C under a 5% CO_2_/95% air atmosphere.

### Viruses

A recombinant HeV (rHeV) was used in which the firefly luciferase gene had been inserted into the HeV genome between the P and M genes. This luciferase gene is expressed to high levels during virus replication and does not result in significant virus attenuation or reduction of CPE *in vitro* [17].

A clinical isolate of HeV (Hendra virus/Australia/Horse/2008/Redlands) was isolated in Vero cells from the spleen of a horse infected in the Redlands Bay outbreak in July 2008 and was passaged three times in Vero cells [32].

### Transfection and Infection

HeLa cells were cultured to 90% confluence in 96 or 24 well plates for 24 hours before transfection. Cells were transfected for 4 hours in OptiMem serum free media (Invitrogen, Carlsbad, USA) using Oligofectamine (Invitrogen) according to the manufacturers’ guidelines. Under BSL4 conditions, transfection medium was removed from the plates and the virus inoculum diluted in EMEM growth medium containing penicillin (100 U/ml), streptomycin (100 µg/ml) and 10% FCS was added to triplicate wells in volumes of 100 µl. Plates were incubated for at 37°C for 24 hours.

For rHeV-luciferase infection, 50 µl of Bright Glo luciferase reagent (Promega) was added to each well. Plates were incubated at room temperature for 5 minutes and then read using a Synergy H4 microplate reader (Biotek Instruments Inc). The read out was relative light units/well.

For clinical HeV infection, the culture medium was stored at −80°C for titration and TCID_50_ calculation. Live HeV in cells was inactivated with 150 µl cell lysis buffer (RLT, Qiagen) for RNA extraction, or immersed in paraformaldehyde or ice-cold methanol [33] for immunofluorescence microscopy. Samples were placed in heat sealed plastic bags and the bags surface was sterilised with Microchem Plus during removal from the BSL4 laboratory.

### TCID_50_ Analysis

10-fold dilutions of tissue culture supernatants were made in media and Vero cells added (5×10^4^ cells/well) in a 96-well tissue culture. Plates were incubated for 3 days at 37°C, 5% CO2 and scored for cytopathic effect. The infectious titre was calculated by the method of Reed and Muench [34].

### qRT-PCR

RNA from samples inactivated with RLT was purified by using the RNeasy mini kit (Qiagen). 0.5–2 µg total RNA was used for reverse transcription (RT) and quantitative real time PCR (qRT-PCR) using a Power SYBR Green RNA-to-C_T_ 1-Step kit as per the manufacturer’s instructions (Applied Biosystems). Quantitative PCR was performed on an ABI Prism 7700 Sequence Detection System (Applied Biosystems). The comparative threshold cycle (Ct) method was used to derive fold change gene expression. Gene of interest primers were specific for HeV N, 5′-GATACAGCCGAGGAAAGTGA-3′ (sense) and 5′-CCTCATCTCTGTCAGCCATT-3′ (antisense), while reference gene primers were specific for human Lamin A/C [35].

### HeV Immunofluorescence Microscopy

For immunofluorescence labelling and confocal analysis, cells were seeded onto 13 mm glass coverslips (Grale Scientific, Victoria, Australia) in 24-well plates. They were allowed to attach overnight and transfected/infected as described above. Cells were fixed for 40 min in 4% paraformaldehyde in phosphate buffered saline (PBS) and stored at 4°C in PBS. Cells transfected with individual siRNAs against the HeV proteins were immunolabelled with antibodies recognising the appropriate expressed protein. Fixed cells were permeabilised with 0.1% triton X-100 (Sigma) in PBS, washed with PBS and non-specific binding blocked with 30 min incubation in PBS containing 0.5% bovine serum albumin (PBS/BSA). Primary and secondary antibodies were diluted in PBS/BSA and incubated for 60 min. Unbound primary antibody was removed with 3×5 min PBS washes and bound antibody was detected with species specific Alexa Fluor 488 conjugated secondary antibodies (Life Technologies). Following 3×5 min washes the nuclei were labelled with a 1∶4000 dilution of DAPI (Sigma) in dH_2_O. Coverslips were mounted in Vectashield (Vector Laboratories, Abacus, Brisbane) and sealed with nail varnish. They were imaged in a Leica (Leica Microsystems, Sydney) SP5 confocal microscope. Microscope settings were the same for all images.

For immunofluorescence labelling and microscopy analysis, HeLa cells were seeded in 24 well plates and transfected/infected as described above. Cells were fixed in ice-cold methanol and blocked for half an hour at room temperature in 1% BSA in TBS-T. Primary antibody was incubated overnight at 4°C (Rabbit anti-HeV-N antibody). Plate was washed three times and secondary antibody (anti-rabbit Alexa Fluor 488 conjugate antibody) with DAPI nuclear stain was incubated for half an hour. Plates were subsequently washed three times and imaged in an EVOS Microscope (Advanced microscopy group, Seattle, USA). Microscope settings were the same for all images.

### Statistics

The difference between two groups was statistically analysed by Student’s *t*-test. P-values <0.05 were considered to be statistically significant.
